# MiRNAs of peripheral blood as the biomarker of schizophrenia

**DOI:** 10.1186/s41065-017-0044-2

**Published:** 2017-08-29

**Authors:** Kuanjun He, Chuang Guo, Lin He, Yongyong Shi

**Affiliations:** 1 College of Life Science, Inner Mongolia University for Nationalities, Tongliao, Inner Mongolia 028043 People’s Republic of China; 2 Bio-X Institutes, Key Laboratory for the Genetics of Developmental and Neuropsychiatric Disorders (Ministry of Education), the Collaborative Innovation Center for Brain Science, Shanghai Jiao Tong University, Shanghai, 200030 People’s Republic of China; 3 Shanghai Key Laboratory of Psychotic Disorders, Shanghai Mental Health Center, Shanghai Jiao Tong University School of Medicine, Shanghai, 200030 People’s Republic of China; 4 Institute of Neuropsychiatric Science and Systems Biological Medicine, Shanghai Jiao Tong University, Shanghai, 200042 People’s Republic of China

**Keywords:** Schizophrenia, Biomarkers, Peripheral MiRNAs, Significance and values

## Abstract

The diagnosis of schizophrenia is currently based on the symptoms and bodily signs rather than on the pathological and physiological markers of the patient. In the search for new molecular targeted therapy medicines, and recurrence of early-warning indicators have become the major focus of contemporary research, because they improve diagnostic accuracy. Biomarkers reflect the physiological, physical and biochemical status of the body, and so have extensive applicability and practical significance. The ascertainment of schizophrenia biomarkers will help diagnose, stratify of disease, and treat of schizophrenia patients. The detection of biomarkers from blood has become a promising area of schizophrenia research. Recently, a series of studies revealed that, MiRNAs play an important role in the genesis of schizophrenia, and their abnormal expressions have the potential to be used as biomarkers of schizophrenia. This article presents and summarizes the value of peripheral blood miRNAs with abnormal expression as the biomarker of schizophrenia.

## Background

Schizophrenia is a kind of brain dysfunction that can induce cognitive, emotional, mental and behavioral disorders. It affects about 1 ~ 1.5% of the world population [1]_._ Research findings showed that genetic factors were the main causes of schizophrenia and the heritability of schizophrenia can be as high as 80% [2]. It often occurs in young adults with symptoms so subtle that is difficult to identify and diagnose. The psychiatric diagnosis of schizophrenia specified in the latest [5th] edition of *The Diagnostic and Statistical Manual of Mental Disorders* (DSM) is based on symptoms rather than on pathophysiological and biochemical indicators [3]. The main symptoms of schizophrenia can be seen in Table 1. At present, the diagnoses of all mental disorders are based on signs and symptoms, rather than on pathophysiological and biochemical indicators [3]. Psychiatry has thus become one branch of medicine which does not rely on laboratory tests [4].

**Table 1:** The main symptoms of schizophrenia

Main symptons	Explaination
Delusions	These are false beliefs that are not based in reality
Hallucinations	Seeing or hearing things that aren’t there.
Disorganized thinking	Using words and sentences that don’t make sense to others.
Extremely disorganized or abnormal motor behavior	Acting in an odd or repetitive way, like walking in circles or writing all the time, or sitting perfectly still and quiet for hours on end.
Negative symptoms	This refers to reduced or lack of ability to function normally.

Biomarkers are substances that can reflect the physiological and biochemical status of an organism. They serve as indicators for pharmacological response in a normal biological process, case procedure or therapeutic intervention. The blood, urine, cerebrospinal fluid, autopsy specimens and tissue samples of schizophrenia patients provide rich gene and protein expressions under physiological and pathological conditions. A comprehensive analysis of these expressions is helpful in the identification of the biomarkers and in the determination of the therapeutic intervention measures and drug targets in the early diagnosis of the disease [5]. The detection of schizophrenia biomarkers aids prediction of the occurrence of the disease, accurate diagnosis, prognosis and treatment [6].

## MiRNAs and schizophrenia

The etiology of schizophrenia is so complex that its causes are not yet fully understood. Both genetic and environmental factors are involved in the occurrence of schizophrenia [7, 8]. MicroRNAs (miRNAs) are a class of small, endogenous and noncoding single-stranded RNAs, 22–25 nucleotides in length, which participate in the post-transcriptional regulation of gene expression. MiRNAs are estimated to regulate the translation of up to 60% of protein-coding genes [9]. Due to the differential target binding patterns, as many as 200 target genes may be regulated by a single miRNA [10]. It has been found that miRNAs were commonly present in a variety of organisms and are involved in almost all life processes, including development, differentiation, growth regulation and apoptosis [11].

It was confirmed that about 70% of human miRNAs express in the nervous system [12] and so are variously involved in the regulation of neural structure and function. For example, they play a role in the formation of dendrites and dendritic spines in axon growth as well as in neural developments and the maturation process [13]. Studies have demonstrated that they are also involved in the occurrence of neuropsychiatric disorders, and that their abnormal expressions could be treated as potential biomarkers [14, 15]. Miller et al. [14] (2010) and Sun [15] (2015) held the view that changes of miRNA expressions played a role in the genetic and biological mechanisms of neuropsychiatric diseases.

Epidemiological evidence suggests the etiology of schizophrenia may involve both genetic and environment factors. Thus, the environmental contribution to schizophrenia progression is through epigenetic mechanisms [16]. The study of mental illness from the perspective of epigenetics therefore informs and illuminates its pathogenesis. In the biological pathways of miRNA, the variation of miRNA target genes may also play a role in the occurrence of primarily schizophrenia. Beveridge et al. [17] (2012) considered that miRNAs influenced the occurrence of schizophrenia via the development of the central nervous system, gene expression regulation and other mechanisms. Since miRNAs are extensively regulated by transcription and are sensitive to changes in the biological pathway, miRNA abnormalities or mutations in cell may lead to neurological disorders including the pathophysiological changes of schizophrenia. Guo et al. [18] (2010) confirmed that miR-195 was involved in a complex regulatory network by which the signaling pathways of the occurrence of schizophrenia were affected. The gene encoding miR-346 was located at the intron glutamate receptors ionic δ1 gene (*GRID1*), and the GRID1 gene is the susceptibility gene for schizophrenia [19]. MiR-30a-5p and miR-195 may regulate the gene expression of brain-derived neurotrophic factors (*BDNF*), so it is suggested that miR-195 may be involved in the pathogenesis of schizophrenia by regulating BDNF [20].

GWAS studies verify that rs1625579 located within miR-137 genes (1p21.3) was significantly associated with schizophrenia (*P* = 1.6 × 10^−11^) [21]. The regulation target genes of miR-137 are CUB and Sushi multiple domains 1 gene (*CSMD1*), WW domain binding protein 1 like gene (*C10orf26*), calcium voltage-gated channel subunit alpha1 C gene (*CACNA1C*), transcription factor 4 gene (*TCF4*) and zinc finger protein 804A *(ZNF804A*), and these genes were confirmed to be genetic risk genes for schizophrenia [22–25].

Through bioinformatics and functional test methods, Valles et al. [26] (2014) confirmed again that five target genes that miR-137 regulated, including *CACNA1C, TCF4* and *ZNF804A*, belong to the glucocorticoid receptor-dependent signal transduction network. *CACNA1C* gene in particularly has been repeatedly shown to be genetic susceptibility genes for schizophrenia in different types of population [21, 22, 27, 28].

## MiRNAs from peripheral blood as potential biomarkers for schizophrenia

Many miRNAs are specifically expressed in cells or tissues, and their expression levels are also related to the pathological or physiological processes of the corresponding cells or tissues. Abnormal expression of miRNAs reflects the pathological state of the organism. Studies have found that the expression profiles of some miRNAs were significant different between the patient and the normal. Differentially expressed miRNAs are likely to be a noninvasive and accurate new biomarker for the diagnosis of disease. Although the etiology of schizophrenia is not certain, abnormal expressions of miRNAs are detected in brain tissues and peripheral blood on the part of schizophrenia patients. As biomarkers of tumors, miRNAs have been shown to be beneficial in clinical stratification, and to have even greater predictive value compared with mRNA [17]. The abnormally expressed miRNAs have been recently isolated from brain tissue [29–31], whole blood [32, 33], serum [34], plasma [35, 36] and PBMC [37], and recognized as potential biomarkers in the diagnosis of schizophrenia. The difficulties of conducting brain biopsies have consequences for obtaining abnormally expressed miRNAs for the purpose of prediction and diagnosis of a neurodegenerative disorder [38]. Since the expression profiles of miRNAs that are present in peripheral blood change with the changes of the body’s physiological and pathological conditions [39], they have important clinical applications. Research investigating biomarkers of schizophrenia in peripheral blood is increasing. Particularly, more and more molecular detection techniques with high specificity and sensitivity are applied in the search for serum markers or genetic markers in peripheral blood.

Abnormally expressed miRNA has also been detected in the peripheral blood mononuclear cells and the plasma of the patients with schizophrenia. Gardiner et al. [37] (2011) analyzed miRNAs in peripheral blood mononuclear cells from 112 psychiatric patients and 76 non-psychotic patients under symptom control. They found that the 83 miRNAs expression in the 112 psychiatric patients was significantly lower than that of the non-psychotic group. 17 down-regulated miRNAs were from the imprinting area, DLK-DIO3 region of 14q32, which is closely associated with schizophrenia. Using qRT-PCR, 7 miRNAs (miR-31, miR-431, miR-433, miR-107, miR-134, miR-99b and miR-487b) were confirmed again to down-regulate.

Using microarray, Lai et al. [40] (2011) compared 30 schizophrenia patients with 30 controls and discovered differences in the miRNA expression of peripheral blood mononuclear leukocytes. Logistic regression analysis of the expression differences in the 7 miRNAs (hsa-miR-34a, miR-449a, miR-564, miR-432, miR-548d, miR-572 and miR-652), revealed emotional retardation, language poverty and other neurocognitive dysfunction-associated symptoms. Shi et al. [34] (2012) found that miR-181b, miR-219-2-3p, miR-1308 and let-7 g in serum were up-regulated, while miRNA-195 was down-regulated. De la Morena et al. [32] (2013) investigated the miRNA expressions in the peripheral blood of patients with 22q11.2DS (22q11 micro-deletion syndrome), and found that 18 miRNAs were differently expressed, and that the miR-185 in the neurons of 90% to 95% of the 22q11.2DS patients was down-regulated.

By using real-time quantitative PCR, Sun et al. [35] (2014) analyzed the expression profiles of nine schizophrenia-related miRNAs in peripheral blood plasma and mononuclear cells, and confirmed that miRNA30e specificity in plasma could be used as a biomarker for diagnosis of schizophrenia. Wei et al. [41] (2015) demonstrated by using high throughput sequencing and RT- qPCR assays that miR-130b and miR-193a-3p serve as biomarkers for the diagnosis of schizophrenia. Using qPCR, Sun et al. [36] (2015) analyzed the expression profiles of 10 miRNAs in plasma from 61 patients with schizophrenia, 62 normal controls, and 25 patients taking antipsychotic drugs for 6 weeks. It was found that miR-30e, MiR-181b, miR-34a, miR-346 and miR-7 as a whole can be used as a non-invasive diagnostic marker for schizophrenia, and that miR-132, miR-181b, miR-30e and miR-432 can all be used as indicators of schizophrenia symptom improvement, drug efficacy and prognosis.

Using qPCR, Lai et al. [33] (2016) analyzed the expressions of 7 miRNAs (hsa-miR-34a, miR-449a, miR-564, miR-432, miR-548d, miR-572, miR-542 and MiR-652) that had previously been identified as potential biomarkers for schizophrenia in acute disease. After 2 months of hospitalization and symptomatic relief, the expressions of the 7 miRNAs in peripheral blood of patients did not change and that their expressions in peripheral blood were stable as schizophrenia diagnostic biomarkers. Using RT-qPCR technology, Liu et al. [42] (2017), suggested that the EGR1-miR-30a-5p-NEUROD1 axis might serve as a promising biomarker for diagnosis and treatment monitoring for patients in an acute psychotic state.

The above tests confirm that miRNA profiling in peripheral blood can be used to determine biomarkers for schizophrenia and related subtypes of mental illness. MiRNAs of peripheral blood that can be biomarkers of schizophrenia can be seen in Table 2. It may help to elucidate the etiology of schizophrenia with ambiguous clinical symptoms and identifies potential differences between psychiatric disorders with similar, easily confusable symptoms [43].

**Table 2:** The list of miRNAs of peripheral blood that can be used as biomarkers of schizophrenia

MiRNA	Biological functionality	Direction associated with schizophrenia	As Biomarkers	Author (Refs.)
miRNA30e	Mitosis, cell division, etc	up-regulation	hsa-miRNA30e	Sun et al. [35] (2014)
miR-130b	nervous system development, neuron differentiation, etc	up-regulation	hsa-miR-130b	Wei et al. [41](2015)
miR-193a-3p	neuron migration, nervous system development, etc	up-regulation	hsa-miR-193a-3p	
miR-30e	as above	up-regulation	hsa-miR-30e, hsa-miR-181b, hsa-miR-34a, hsa-miR-346 and hsa-miR-7 as a whole	Sun et al. [36] (2015)
miR-181b	central nervous system development, aging, etc	up-regulation		
miR-34a	neuron migration, generation of neurons, organ regeneration, etc	up-regulation		
miR-346	response to stress, nervous system development, learning or memory, etc	up-regulation		
miR-7	dendrite development, cellular response to stress, etc	up-regulation		
miR-34a	as above	up-regulation	hsa-miR-34a	Lai et al. [33] (2016)
miR-449a	neuron apoptotic process, cellular response to hypoxia, etc	up-regulation	hsa-miR-449a	
miR-564	PI3K signaling networks, MAPK signaling pathway, etc	up-regulation	hsa-miR-564	
miR-432	PI3K/AKT/mTOR signaling pathway, cell proliferation and differentiation, etc	up-regulation	hsa-miR-432	
miR-548d	nervous system development, positive regulation of rho gtpase activity, etc	up-regulation	hsa-miR-548d	
miR-572	cellular response to extracellular stimulus, organ regeneration, response to hyperoxia, etc	up-regulation	hsa-miR-572	
miR-652	regulation of energy homeostasis, dendrite morphogenesis, etc	up-regulation	hsa-miR-652	
miR-30a-5p	response to hypoxia, neuron migration, immune response, etc	down regulation	EGR1-miR-30a-5p-NEUROD1 axis	Liu et al. [42] (2017)

## Conclusions

Progress in the foregoing highlights the potential significance of miRNA as a diagnostic marker of schizophrenia. MiRNA expression profiles from peripheral blood not only are potential schizophrenia biomarkers but also have association with schizophrenia subtypes. The study of miRNA expression profiles in peripheral blood is of far reaching significance because it provides the basis for the early detection of schizophrenia, disease stratification and the prediction of drug response and side effects. It enables further study of the regulatory mechanisms of gene expression, their relationship with the disease, their proteins expression profiles, and the molecular mechanisms involved in the pathogenesis of schizophrenia, and it will lead to the elucidation of the complex etiology of schizophrenia. Studies of the molecular mechanisms of the occurrence and development of schizophrenia, its specificity, especially of the early warning and diagnostic biomarkers obtained directly from the blood, have been an important and urgent priority in schizophrenia research. Ongoing improvements in the detection, assessment and intervention strategies for miRNA are promoting the development of the diagnosis and treatment of schizophrenia, and facilitating miRNA applications in other disease diagnoses and treatments.

## Abbreviations

22q11DS: 22q11 micro-deletion syndrome
*BDNF*: Brain-derived neurotrophic factors
*C10orf26*: WW domain binding protein 1 like gene
*CACNA1C*: Calcium voltage-gated channel subunit alpha1 C gene
*CSMD1*: CUB and sushi multiple domains 1 gene
DSM: The diagnostic and statistical manual of mental disorders
*GRID1*: Intron glutamate receptors ionic δ1 gene
MiRNAs: MicroRNAs
*TCF4*: Transcription factor 4
*ZNF804A*: Gene and zinc finger protein 804A
